# The Effects of a Parent-Implemented Language Intervention on Late-Talkers’ Expressive Skills: The Mediational Role of Parental Speech Contingency and Dialogic Reading Abilities

**DOI:** 10.3389/fpsyg.2021.723366

**Published:** 2021-09-09

**Authors:** Chiara Suttora, Mariagrazia Zuccarini, Arianna Aceti, Luigi Corvaglia, Annalisa Guarini, Alessandra Sansavini

**Affiliations:** ^1^Department of Psychology “Renzo Canestrari”, University of Bologna, Bologna, Italy; ^2^Department of Medical and Surgical Sciences, University of Bologna, Bologna, Italy; ^3^Neonatology and Neonatal Intensive Care Unit, S. Orsola-Malpighi Hospital, Bologna, Italy

**Keywords:** parent-implemented intervention, child-directed speech, expressive language delay, late-talkers, preterm birth

## Abstract

Several qualitative and quantitative features of parental speech input support children’s language development and may play a critical role in improving such process in late talkers. Parent-implemented interventions targeting late-talkers have been developed to promote children’s language outcomes by enhancing their linguistic environment, i.e., parental speech input. This study investigated the effect of a parent-implemented intervention in increasing late talkers’ expressive skills through modifications in structural and functional features of parental speech input. Forty-six thirty-one-month-old late talkers differing in their birth condition (either low-risk preterm or full-term) participated in the study with a parent; 24 parent-child dyads received a parent-implemented intervention centered on dialogic reading and focused stimulation techniques, whereas the other 22 dyads constituted the control group. At pre- and post-intervention, dyads took part in a parent-child shared book-reading session and both parental and child’s speech measures were collected and examined. Results showed that the intervention positively affected parents’ use of responses and expansions of children’s verbal initiatives, as well as the parental amount of talking over reading, whereas no structural features of parental input resulted modified. Mediation analyses pointed out that the intervention indirectly enhanced late-talkers’ use of verbal types and tokens through changes in parental use of expansions and amount of talking over reading. As birth status was entered as a covariate in the analysis, these findings can be extended to children with different gestational age. We conclude that the parent-implemented intervention was effective in supporting late-talkers’ gains in language development as a cascade result of the improvements in parental contingency and dialogic reading abilities. These promising findings suggest to examine not only children and parental outcomes but also the intervention mechanisms promoting changes in late-talkers’ language development as a clearer view on such process can inform the development of feasible, ecological and effective programs.

## Introduction

### Relationship Between Parental Speech and Child Language Development

The first 1,000 days of life are considered a fundamental time window in which children’s developmental trajectories and future outcomes are shaped. Within this period, providing children with nurturing experiences such as responsive caregiving and adequate learning opportunities is vital (Britto et al., 2017). Language stimulation by parents and caregivers is one of these essential nurturing experiences (Golinkoff et al., 2019). Parents usually talk to their infants and children using a particular speech register also known as infant- or child-directed speech (IDS, CDS). Such input has specific prosodic (i.e., pitch, length of sounds, intensity), structural (i.e., quantitative aspects of speech, lexical and syntactic complexity), and functional features (i.e., directiveness, contingency, and tutorial function of parental utterances directed at the child) that make it an optimal input for toddlers developing language; child-directed parental utterances are typically high pitched and modulated, short in their length, built with a simple and redundant lexicon, and contingent to child’s communicative bids (Tamis-LeMonda et al., 2001; Hoff and Naigles, 2002; Huttenlocher et al., 2002; Rowe, 2012).

Differences in structural and functional features of parental speech relate to variability in child language learning (Anderson et al., 2021). Concerning structural features, much research on parental input emphasizes the role of input quantity in predicting both children’s rate of vocabulary growth and vocabulary skills (Huttenlocher et al., 1991, 2010; Hart and Risley, 1995; Weisleder and Fernald, 2013). Of no lesser importance, input quality, often calculated as word types and input complexity, expressed through the mean length of utterance index (MLU), also accounts for variability in children’s lexical outcomes (Hoff and Naigles, 2002; Pan et al., 2005; Huttenlocher et al., 2010; Rowe, 2012; Anderson et al., 2021). Moreover, a very recent study (Silvey et al., 2021) indicates that not the absolute complexity of syntactic input captured in a specific time of development but the extent to which input complexity increases over time predicts children’s grammatical outcomes.

Functional features of parental input are also thought to contribute to child language development. Parental ability to respond contingently to children’s attentive focus and communicative initiatives is one of these features, with literature findings showing that differences in maternal contingent responding predict children’s vocabulary growth (Tamis-LeMonda et al., 2001, 2014). A relevant role in determining children’s linguistic outcomes is also played by parental recasts or reformulations of children’s linguistic attempts which encompass all those responses in which parents imitate, expand, or reduce children’s original verbal utterances (Taumoepeau, 2016). These reformulations are not only inherently contingent to children’s initiative but provide them with relevant lexical and syntactic data. Parental repetitions draw a child’s attention to his/her own verbal production, allowing a phonological comparison with the adult form; moreover, expansions provide the child with further relevant lexical and syntactic data linked to the original verbal production, exposing him/her to new learning opportunities. The role of structural and functional features of parental input addressed to children with delayed language development has also been investigated (Girolametto et al., 1999, 2002; Vigil et al., 2005; D’Odorico and Jacob, 2006; Levickis et al., 2018; Suttora et al., 2020). The term “late talkers” refers to those children who lag behind in several aspects of language, showing a slower rate of growth in language learning and limited expressive vocabulary (i.e., below the 10th percentile with respect to normative data), in absence of sensory, cognitive or socio-emotional difficulties (Hawa and Spanoudis, 2014). As late talkers represent a significant proportion of 2–3-year-old children—with prevalence ranging from 9 to 21% (Reilly et al., 2007; Korpilahti et al., 2016; Sansavini et al., 2021)—it is relevant from a clinical stance to describe the peculiarities of their linguistic milieu to capture which aspects of parental input could be enhanced and/or modified. With respect to the structural features of parental input, literature addressing late talkers’ samples are coherent in finding no significant differences in terms of input quantity (i.e., lexical rate), quality (i.e., lexical diversity), and complexity (i.e., MLU) when this input is compared with that addressed to typically developing children (Paul and Elwood, 1991; Vigil et al., 2005; D’Odorico and Jacob, 2006; Suttora et al., 2020). However, an input characterized by high levels of grammatical complexity, lexical rate, and diversity has been linked to lower abilities in late talkers’ spontaneous and reported lexical production (Girolametto et al., 1999; Suttora et al., 2020). According to Girolametto et al. (1999), this latter pattern of associations might be representative of an “idiosyncratic feedback loop,” a circle in which children’s linguistic impairment negatively affects their parents’ input, which in turn constitutes a further complication for children’s language improvement. In light of this, regardless of differences in input quality and/or quantity, late talkers could benefit from a less complex input characterized by shorter, simpler, and clearer utterances. As for the functional features of parental input, literature suggests that parents of late talkers are less contingent to their children displaying fewer responses to their children’s initiations and fewer expansions than parents talking to their children with typical language skills (Vigil et al., 2005). Again, lower use of expansions in the input directed to late talkers predicts smaller vocabulary and expressive skills (Girolametto et al., 1999, 2002), also when assessed at 2-year distance (Levickis et al., 2018). According to these findings, interventions aimed at enhancing late talkers’ linguistics environment, by improving parental responsiveness and expansions while keeping its complexity adjusted to children’s communicative skills, might constitute a privileged route to support children with language delays.

### Parent-Implemented Interventions Supporting Late-Talkers’ Language Development

Treatment options for late talkers include both direct and indirect interventions. The formers consist of individual treatment delivered by a speech-language therapist (SLT) in a clinical setting and may also involve parents who can be asked to do at-home activities with their child to support the treatment. The efficacy of direct interventions with SLTs is demonstrated by a Cochrane review considering studies involving children with phonological and lexical difficulties (Law et al., 2017).

Indirect interventions are programs in which parents—trained, guided, or supported by SLTs and/or psychologists with speech-language expertise—are the main providers of the treatment. Parent training can be individual or group based. As previously accounted, given the critical role of caregivers in supporting and enhancing their children’s language development and the differences highlighted in parental speech directed at children with delayed language acquisition, programs designed to train caregivers how to best support language development are relevant components of effective intervention practices. In this direction, studies aimed at comparing directed versus parent-implemented interventions for late talkers revealed a lack of differences in their efficacy in enhancing children language skills (Roberts and Kaiser, 2011; DeVeney et al., 2017; Tosh et al., 2017) making parent-implemented interventions a valid option for early intervention. In these programs, parents are trained to use specific language and conversational strategies aimed at supporting their children’s language learning by enhancing their linguistic environment. Specifically, parents are taught: (a) to follow children’s attention and lead during conversation trying to get them focused on the exchange; (b) to increase their responsiveness to children’s communicative and verbal initiatives, by recasting, imitating, and expanding their verbal productions; and (c) to limit an excessive use of questioning and/or directiveness in the input they address to them. These interventions can also include parent training on focused stimulation and dialogic book reading. In the first technique, parents are trained to repetitively use few selected target words during play or routine contexts (Girolametto et al., 1996a). In the second, parents are taught how to elicit conversation and turn-taking during a book sharing activity (Buschmann et al., 2009). Among parent-intervention programs, the Hanen Parent Programs (HPP; Manolson, 1992) is one of the most common, directed not only at children with primary language difficulties, but also at children with secondary linguistic issues, such as children with motor disorders, cerebral palsy, or autism spectrum disorder (Pennington and Thomson, 2007; Weitzman, 2013). In HPP caregivers are instructed on how to follow their children’s attentional states and how to use specific responsive interaction strategies aimed at supporting children’s language learning throughout daily routines. Summarizing, parent-implemented interventions aim at affecting late talkers’ language skills through a cascading effect, i.e., because of modifications in their parents’ input and conversational strategies (Roberts and Kaiser, 2012).

The efficacy of parent-implemented programs on late talkers’ language outcomes is consistent and well-documented. Roberts and Kaiser’s meta-analysis (Roberts and Kaiser, 2011) of 18 studies indicated that children participating to parent-implemented interventions scored better than controls in almost all measures of language development—observed and parent-reported—with greater effect sizes for measures of expressive morphosyntax and receptive vocabulary. These findings were confirmed even when entering intellectual disability as a moderator, as seven out of eighteen studies included in the meta-analysis involved children with cognitive disabilities, genetic syndromes, or autism. Narrowing their analysis to studies addressing children with language delay, Tosh et al.’s (2017) review reported very similar conclusions confirming that children enrolled in parent-implemented programs showed more favorable language outcomes than children in the control conditions. Finally, Heidlage et al.’s (2020) meta-analysis on 25 RCT studies indicated that, on average, parent-implemented language interventions have significant effects on children’s expressive vocabulary, both when interventions focus on caregiver-child play routines and on book sharing activities.

As regards the effects of parent-implemented programs on caregivers’ input and use of conversational strategies, literature findings are fewer and less clear. Roberts and Kaiser’s (2011) meta-analysis concluded that parent-implemented programs positively impacted caregivers’ outcomes, with particular regard to their responsiveness to children’s communicative initiatives. However, among the studies examined, only four studies addressed interventions directed at parents of children with language delay (Girolametto et al., 1996a,b; Law et al., 1999; Baxendale and Hesketh, 2003). In Girolametto et al. (1996a), mothers in the intervention group significantly produced fewer words per minute and shorter utterances than mothers in the control group at post-intervention assessment, demonstrating an adjustment to children’s communicative level. These mothers also showed greater use of focused stimulation on target words, which was one of the techniques modeled by the intervention. Baxendale and Hesketh (2003), by contrast, found no differences between parents in a HHP group and parents enrolled in conventional clinic therapy group in the use of expansions and imitation strategies, as for all participants there was a significant increase in the use of strategies such as imitation and expansion from pre- to post-intervention. Law et al. (1999) also failed to find significant effects of the intervention on parental outcomes.

More recent findings shed some light on the effects of parent-implemented interventions on caregivers’ input and strategies. Heidlage et al.’s (2020) meta-analysis confirmed a significant effect of parent-implemented interventions on parents’ responsiveness but considered this finding as preliminary as it was based on just five studies. Comparing the parent-training Enhanced Milieu Teaching (EMT) to usual care in a sample of 97 parent-child dyads, Roberts and Kaiser (2015) found that caregivers in the EMT group improved in all language facilitation strategies targeted in the intervention, namely the use of turn-taking, responsiveness, expansion and prompting as assessed during a 20 min play-based caregiver-child interaction. Similar results were underscored by Kruythoff-Broekman et al. (2019) comparing the Target Word program (part of the HHP procedure) to a usual-care control group. At 6-month post-intervention, parental language strategies, as measured with a rating scale during a 5-min parent-child interaction, resulted significantly improved, with an increase in the use of interactive strategies and a decrease of parental utterances aimed at putting pressure on the child. Additional results of Kruythoff-Broekman et al.’s (2019) study revealed that children whose parents reduced pressing behaviors significantly improved their expressive vocabulary and expressive syntax, suggesting a cascading effect of the modifications observed in parental input on children’s gains in language development.

In short, although effects of parent-implemented interventions have been documented both on children linguistic outcomes and, to a lesser extent, on parental input and strategies, studies expressly addressing the effects of such interventions on children’s gains in language skills through modifications in caregivers’ use of input and conversational strategies have not been performed yet. This is our study’s main intent.

### Aims of the Study

The present study aimed at investigating the effect of a parent-implemented language intervention in enhancing structural and functional features of parental communicative input to their own late talking children in the third year of life and eventually triggering positive cascading effects on children’s lexical and grammatical skills.

Firstly, we investigated whether the intervention based on dialogic book reading impacted: (a) structural features of parent speech, such as lexical diversity, rate, and grammatical complexity; (b) functional features of parental input, such as the ability to respond contingently to their own child’s verbal initiatives—by reformulating child’s speech productions—and to engage the child in a conversation during the book sharing activity. As the intervention was mainly focused on promoting functional features of parental conversation, we expected to find a more significant impact of the intervention on these features rather than on structural ones (i.e., lexical diversity, rate, and grammatical complexity).

Secondly, we investigated the effects of the intervention on children’s advances in language development. A significant increase of expressive lexicon in children’s spontaneous speech, as regards lexical and grammatical measures, was expected as suggested by previous works that documented the efficacy of the same intervention on measures of children’s lexical and grammatical skills collected through parental reports (Bello et al., 2019; Zuccarini et al., 2020). We hypothesized that this effect was triggered by parental input improvement determined by the intervention. As the intervention mainly addressed functional features of parental input, we expected that significant changes in these features would, in turn, positively impact on child’s language development. As the intervention was provided to a group of children differing for birth condition (i.e., low-risk preterm and full-term) this variable was controlled in our analyses.

## Materials and Methods

### Participants

Sixty-two parents with their late-talking children were invited to participate in the study. Fifty-nine out of them accepted to be enrolled in the study. Criteria of inclusion in the study consisted in children being monolingual or mainly exposed to the Italian language from birth onward, being either full-term (i.e., with a gestational age ≥ 37 weeks) or low-risk preterm (i.e., with a gestational age < 37 weeks) and not having any severe neurological impairment and/or congenital malformations, visual, hearing, or motor impairments, or severe neonatal complications, or severe cognitive deficits (Bayley-III cognitive score < 70).

With a convenience sample methodology parents were asked whether they would participate in the intervention condition. Thirty-one parents accepted to participate and 28 declined the invitation and were assigned to the control condition. Parental speech to the child and child spontaneous vocal productions were assessed during two assessments conducted at the Developmental Psychology Lab at the University of Bologna when children were around 31-month-old (*M* = 31.13, *SD* = 1.20)—pre-intervention assessment—and 37-month-old (*M* = 37.06, *SD* = 1.47)—post-intervention assessment. The parent-implemented language intervention lasted approximately 2 months, between the pre- and post-assessment. Eleven participants, 6 in the intervention and 5 in the control group, did not attend or complete the post-intervention assessment and were thus excluded from the data analysis. Other 2 dyads, one from the intervention and one from the control group, were also excluded as the parent who attended the pre and the post-intervention was different. Thus, the final sample consisted of 46 parents and their children with 24 parents participating in the intervention and 22 included in the control group. A flow diagram provides an overview of parents participating in the study (see Supplementary Figure 1).

The final sample included 17 parents of low-risk preterm children, born before 37 weeks of gestation, at the Sant’Orsola-Malpighi Hospital of the University of Bologna. Perinatal characteristics of the subgroup of low-risk preterm children are reported in Supplementary Table 1. The remaining participants (*n* = 29) were parents of healthy full-term children that were born in the same hospital. Parents of low-risk preterm and full-term children were not equally distributed in the intervention and control groups with proportionally more parents of low-risk preterm children participating in the intervention (intervention *n* = 12; control *n* = 5) compared to parents of full-term children (intervention *n* = 12; control *n* = 17), χ^2^(1, *N* = 46) = 3.66, *p* = 0.05.

Biological and sociodemographic characteristics of children and parents in the intervention and control groups are described and compared in Table 1. The same table displays information about the age of children at the pre- and post-intervention assessment, as well as a measure of the time interval between pre- and post-intervention assessment. For children born preterm, age was corrected for weeks of prematurity to consider their level of neurobiological maturation as done in previous studies (Sansavini et al., 2011). Children in the intervention and control groups were similar in mostly all sociodemographic variables, with the only exception of their attendance to child-care centers that was higher for children in the intervention group. With regard to parental variables, mothers in the intervention group were significantly older than mothers in the control group.

**TABLE 1:** Sociodemographic characteristics of participants in the entire sample.

	**Intervention group**	**Control group**		
**Participants’ characteristics**	**(*n* = 24)**	**(*n* = 22)**	**χ^2^/*t* (df)**	***p***
Gestational age (weeks), mean (SD)	37.38 (3.12)	38.03 (2.76)	0.74 (44)	0.464
Birthweight (grams), mean (SD)	2782.58 (942.69)	2939.82 (648.12)	0.64 (44)	0.524
Length of stay in hospital (days), mean (SD)	13.58 (35.62)	4.36 (5.63)	−1.19 (44)	0.237
Gender (Female), *n* (%)	10 (41.7)	7 (31.8)	0.48 (1, 46)	0.489
Firstborn, *n* (%)	14 (58.3)	8 (36.4)	3.08 (1, 46)	0.214
Twins, *n* (%)	4 (16.7)	4 (18.2)	0.02 (1, 46)	0.892
Otitis media, *n* (%)	1 (4.2)	2 (9.1)	0.46 (1, 46)	0.499
Family history of language and/or learning disorders (LLD), *n* (%)	6 (25.0)	4 (18.2)	0.31 (1, 46)	0.575
Child-care center attendance, *n* (%)	21 (87.5)	13 (59.1)	4.80 (1, 46)	**0.028**
Other parent input besides Italian, *n* (%)	2 (8.3)	1 (4.5)	0.23 (1, 46)	0.632
Mother’s age (years), mean (SD)	40.04 (5.20)	35.98 (4.69)	−2.79 (44)	**0.008**
Father’s age (years), mean (SD)	42.00 (5.09)	38.70 (5.59)	−1.98 (41)	0.055
Mothers with high educational level (>13 years), *n* (%)	17 (70.8)	13 (59.1)	0.70 (1, 46)	0.404
Fathers with high educational level (>13 years), *n* (%)	11 (46.8)	9 (40.9)	0.11 (1, 46)	0.736
Mother’s nationality (Italian), *n* (%)	23 (95.5)	21 (95.8)	0.01 (1, 46)	0.950
Father’s nationality (Italian), *n* (%)	23 (95.5)	21 (95.8)	0.01 (1, 46)	0.950
Age at pre-intervention (months), mean (SD)	30.86 (1.44)	31.30 (1.06)	1.65 (44)	0.250
Age at post-intervention (months), mean (SD)	37.02 (1.44)	37.13 (1.15)	0.29 (44)	0.777
Pre and post-intervention interval (days), mean (SD)	187.29 (52.21)	177.36 (40.75)	−0.71 (44)	0.479

*Significant results are displayed in bold.*

### Procedure and Study Design

Children identified as late talkers—having an expressive vocabulary size at or below the 10th percentile for their age—through the use and normative values of the Italian version of the MacArthur Bates Communicative Development Inventories (MB-CDI), Words and Sentences Complete Form (Caselli et al., 2015; Rinaldi et al., 2019) were invited, around 31 months of age, with their parents at the Developmental Psychology Lab at the University of Bologna for an assessment of their communicative exchanges. The MB-CDI served as a tool to identify children as late-talkers. Dyads were observed and videotaped during a parent-child shared book-reading session during which both partners’ speech was collected. One parent, more often the mother (except for two children whose father participated in the study), was asked to interact with his/her child by sharing two age-appropriate picture books seated at a child-table. Parents included in the intervention condition attended six 2-h intervention sessions with a trained psychologist. To test the effectiveness of the intervention, a pre-post-intervention assessment was used. Thus, parent-child dyads were invited, when children were around 37 months of age, to participate in a second videotaped book reading session. The pre-intervention session lasted on average 10 min (*SD* = 84 s), the post-intervention session approximately 9 min and 54 s (*SD* = 146 s).

### Parent-Administered Intervention Program

A 2-month-lenght parent-administered manualized intervention, named “Oltre il libro” (Girolametto et al., 2017), was used in the study. This is a dialogic book reading program consisting of 6 training sessions, of about 2 h each, directed at small groups of parents, normally 4–6 people per group. The intervention program is theoretically based on the interactive model of language intervention and it aims at fostering children language development by coaching parents in the use of different conversational strategies during book reading. The intervention aims at coaching parents for: (a) fostering turn-taking skills and promoting the use of extra-verbal cues as intonation, rhythm, and gestures; (b) adjusting their speech to their child’s linguistic skills using simple sentences and redundant lexicon; (c) using, besides close-ended questions, open-ended wh-questions (e.g., “where is Anna hidden?” “why is the elephant sad?”); (d) implementing focused stimulation on target words that are already understood but not produced by the child yet; (e) expanding their child’s verbal production (e.g., the child says “elephant” and the parent replies “yes, the elephant is sad as it cannot find a place to draw”).

### Tools

Child’s expressive vocabulary was assessed with the Words and Sentences Complete Form of the Italian MB-CDI (Caselli et al., 2015), that is a valid and reliable tool to investigate child lexical production and grammatical skills, as indicated by its widespread use in clinical contexts and empirical studies (Sansavini et al., 2019; Majorano et al., 2020; Zuccarini et al., 2020).

### Transcription and Coding

Parental speech directed to the child and child spontaneous speech productions observed during the video-recorded sessions were transcribed into CHAT format of the Child Language Data Exchange System (CHILDES, MacWhinney, 2000) by an experienced speech therapist blind to study hypotheses and child’s age. The transcription unit was the utterance that was defined as any speech production, a vocal sound, a single word, or a sequence of words, delimited by a pause, a conversational turn, or a change in the intonation pattern (Craig, 1982). With respect to the child’s speech, a vocal production was considered verbal and transcribed as a word when at least three of the following criteria were met: (a) occurred at least two times; (b) was phonetically similar to the target word; (c) had a specific referent; and (d) was recognized as a word by the parent during the exchange (Vihman and McCune, 1994). All the vocal productions that did not meet these criteria were transcribed in IPA and classified as unintelligible in transcriptions.

### Structural Features of Parental and Child’s Speech

Once transcribed, child-directed parental utterances and child’s speech production were analyzed with the CLAN software and different measures were obtained. Onomatopoeic productions as well as interjections and unintelligible speech were excluded from these analyses. CLAN automated analysis of the transcriptions generated the following indexes of quantity and complexity of parental and child’s speech input: (a) the frequency of word Types as an index of lexical diversity; (b) the frequency of word Tokens as a measure of lexical rate; (c) the mean length of utterances (MLU), i.e., the ratio of words over utterances, as a measure of speech grammatical complexity. Raw frequencies were converted in rate per 10 min to control for session’s length. Finally, to obtain measures of the change between pre- and post-intervention, deltas were computed for the abovementioned indexes of parental and child’s speech by subtracting from values observed at post-intervention those computed at pre-intervention.

### Functional Features of Parental Input

A further analysis of the transcripts was conducted using CHIP, a CLAN software for the automatic coding and analysis of parent-child conversational interactions. CHIP automatically compares pairs of utterances in which the first is considered the source and the following the response. Through this comparison the software creates a series of additional tiers in the transcript in which responses or self-repetitions are examined. In this study, we focused on the adult tiers (*%adu)* in which parent’s responses to child’s utterances are evaluated. As parents can reply to his/her child in more than one turn following child’s speech production, CHIP command searches parental responses within a six utterances window. In the present data the average distance between child’s source and parental responses was low (*M* = 1.08, *SD* = 0.54 at pre-intervention; *M* = 1.36, *SD* = 0.71 at post-intervention). According to the study’s main aims the following indexes were considered: (a) *Total Responses*, i.e., the total number of parental responses to child’s utterances; (b) *Exacts*, i.e., the number of exactly matching responses (e.g., the child says “hat” and the parent replies “hat”); (c) *Reductions*, i.e., the number of responses in which there was an overlap of at least one word in the source and response utterances with deletions but no additions (e.g., the child says “big hat” and the parent replies “hat”; (d) *Expansions*, i.e., the number of responses containing only exact matches and additions (e.g., the child says “hat” and the parent replies “right, the hat!”).

Moreover, a measure of the amount of parental talking over reading was computed by dividing the amount of talking tokens for the sum of parental talking and reading word tokens. A high value in Talking over Reading measure indicated that a parent spent most of the session engaging the child in a conversational exchange instead of reading the available books. To measures changes in functional indexes of parental speech from pre- to post-intervention delta measures were calculated.

### Reliability

The first author of this manuscript, who was blind to the child’s age and birth status, transcribed 27% (12 at pre-intervention and 13 at post-intervention) of the parent-child sessions to establish transcription reliability. Reliability between the two transcribers was high, with a percent interrater agreement equal to 88% on the segmentation of parents’ utterances and of 87% on the transcription of child’s vocal utterances.

Interrater reliability for parental measures was assessed using the Intraclass Correlation Coefficient (*ICC*) with high levels of agreement resulting for all parent’s measures (*ICCs* > 0.85). Interrater agreement on child’s speech coding into intelligible, unintelligible, or mixed utterances was tested computing Cohen’s Kappa which resulted equal to 0.83. Interrater reliability was more than substantial. Regarding child’s linguistic outcomes (i.e., word types, word tokens, MLU), interrater agreement was achieved by calculating the *ICC*, resulting in optimal values with *ICC* = 0.96.

### Statistical Analyses

Analyses were computed using IBM SPSS Statistics 25 and the macro Process for SPSS (Hayes, 2018). Tests were bilateral with a statistical significance set at 0.05. Preliminary analyses of data distribution revealed that most of the study’s variables were not normally distributed (Kolmogorov–Smirnov and Shapiro–Wilk tests, *p*s < 0.01). Therefore, rank transformation was applied to both parental and child measures.

A set of multivariate MANCOVAs was preliminary carried out to verify that at the pre-intervention assessment parental speech and child’s lexical and grammatical measures were comparable between the intervention and the control group. As the presence of low-risk preterm and full-term children differed between groups, birth condition was included as a covariate in the analyses.

To explore the effect of the intervention (intervention vs. control group) on parental structural and functional speech measures and child’s linguistic measures over time (pre-intervention vs. post-intervention), several repeated measure MANCOVAs were conducted, controlling for birth condition.

**FIGURE 1:**
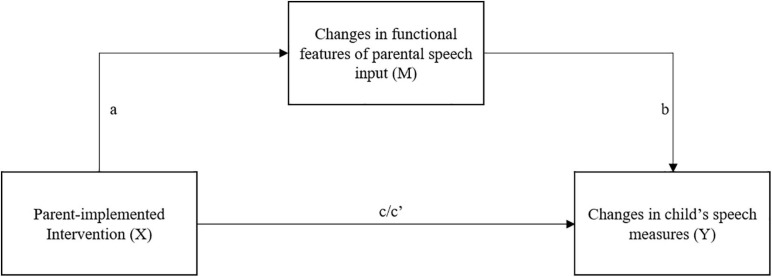
Changes in functional features of parental speech input (M) operate mediationally between Parent-implemented Intervention (X) and Changes in child’s speech measures (Y).

Preliminary Pearson’s correlational analyses were carried out to explore the associations between parental and child’s speech delta measures. Subsequently, indirect effects of the intervention condition on child’s delta speech outcomes through changes in parental speech were tested with mediation analyses using the macro PROCESS, model 4 (Hayes, 2018, p. 585). Unstandardized indirect effects were computed for each of 5,000 bootstrapped samples and the 95% confidence intervals were obtained. In Figure 1 parental changes in speech input is a mediator (M) of the relationship between the parent-implemented language intervention (X) and child’s gain in speech measures from pre- to post-intervention assessment (Y). These latter analyses only included parental measures that resulted significantly affected by the parent-implemented intervention. Again, birth condition was entered in the analyses as a covariate.

### Ethics Statement

The study met ethical guidelines for human subject protections, including adherence to the legal requirements of Italy, and it received formal approval from the Bologna Health Authority’s Independent Ethics Committee (numbers of formal approval documents: EM 194/2017/U_ and EM 193–2018_76/2013/U/Sper/AOUBo). All parents gave informed written consent for study participation, data analysis, and data publication. No incentives or benefits were provided to participants.

## Results

### Pre-intervention Assessment

In Table 2, the descriptive statistics describing parental and child’s speech measures at pre-intervention were reported. No significant differences between parents and children in the control and intervention groups at the pre-intervention assessment were found (for details see Supplementary Table 2).

**TABLE 2:** Means and standard deviations M (SD) for parental and child’s speech measures at pre- and post-intervention assessment, Δ pre-post assessment, and results of repeated measures MANCOVAs performed on pre- and post-intervention data.

	**Pre-intervention assessment**		**Post-intervention assessment**		**Δ Pre-post assessment**		**Univariate test Time X Intervention**		
	**Intervention Group**	**Control Group**	**Intervention Group**	**Control Group**	**Intervention Group**	**Control Group**	***F*(1, 43)**	***p***	**partial η^2^**
	**(*n* = 24)**	**(*n* = 22)**	**(*n* = 24)**	**(*n* = 22)**	**(*n* = 24)**	**(*n* = 22)**			
**Parental speech—structural features**									
Types	202.92 (38.44)	182.32 (47.91)	202.55 (45.31)	186.99 (85.91)	−0.37 (38.86)	4.67 (89.10)	0.03	0.869	0.001
Tokens	603.13 (151.59)	563.55 (201.46)	618.59 (201.59)	521.59 (243.62)	15.46 (152.44)	−41.96 (213.25)	1.17	0.285	0.026
MLU	3.25 (0.42)	3.14 (0.51)	3.47 (0.61)	3.11 (0.38)	0.22 (0.59)	−0.02 (0.37)	2.47	0.124	0.054
**Parental speech—functional features**									
Total Responses	96.92 (55.57)	108.05 (57.69)	137.42 (85.50)	94.77 (79.33)	40.50 (97.30)	−13.27 (85.62)	4.57	**0.038**	0.096
Exacts	3.63 (3.69)	2.95 (3.40)	5.00 (3.99)	3.95 (4.20)	1.38 (4.19)	1.00 (3.52)	0.87	0.356	0.020
Reductions	0.29 (0.69)	0.60 (1.25)	1.25 (1.80)	0.59 (1.09)	0.96 (2.01)	0.00 (1.77)	0.01	0.975	0.001
Expansions	4.29 (4.92)	2.64 (3.26)	9.63 (6.78)	4.68 (5.35)	5.33 (8.19)	2.05 (5.24)	4.06	**0.050**	0.086
Talking over reading	0.69 (0.14)	0.71 (0.17)	0.75 (0.20)	0.62 (0.26)	0.05 (0.20)	−0.09 (0.23)	6.14	**0.017**	0.125
**Child’s speech**									
Types	9.28 (9.09)	6.04 (4.95)	26.29 (21.71)	15.79 (19.69)	17.02 (23.81)	9.75 (16.55)	3.34	0.075	0.072
Tokens	6.11 (8.22)	10.17 (10.84)	56.60 (40.13)	50.48 (78.97)	50.49 (41.92)	40.31 (76.44)	7.41	**0.009**	0.147
MLU	1.05 (0.26)	0.98 (0.33)	1.36 (0.38)	1.03 (0.50)	0.31 (0.42)	0.05 (0.50)	3.25	0.079	0.070

*Significant results are highlighted in bold. Parental and child types and tokens are computed as rate per 10 min.*

### Effects of the Parent-Implemented Intervention on Parental Speech Outcomes

Table 2 summarized the descriptive statistics of parental speech outcomes at pre- and post-intervention assessment. Regarding parent’s speech structural features (i.e., word types, tokens and MLU) no significant effects of intervention were found with the multivariate test indicating a lack of significant effect [*F*_(3, 41)_ = 1.65, *p* = 0.192, partial η^2^ = 0.108]. Regarding parental speech functional features, the multivariate analysis yielded a significant effect [*F*_(5, 39)_ = 2.47, *p* = 0.048, partial η^2^ = 0.241], with univariate results showing that the intervention significantly influenced *Total responses, Expansions, and Talking over Reading* measures (see Table 2). Parents that participated in the intervention showed a significant increase from pre- to post-assessment in the total responses to the child’s verbal initiatives and in the use of utterances aimed to expand the child’s productions when compared to the control group. Furthermore, in the intervention group, a significant increase in the amount of talking over reading was observed from pre- to post-intervention assessment.

### Effects of the Parent-Implemented Intervention on Child’s Spontaneous Speech

The impact of the parent-implemented intervention on child’s spontaneous speech outcomes resulted close to statistical significance with the multivariate test. *F*_(3, 41)_ = 2.61, *p* = 0.064, partial η^2^ = 0.160. Considering the univariate results reported in Table 2, the intervention significantly improved children’s production of word tokens from the pre- to the post-intervention assessment. Also, child’s production of word types and MLU were observed to increase due to the intervention but with *p-*values implying trends to statistical significance.

### Direct and Indirect Effects of the Parent-Implemented Language Intervention on Child’s Speech Outcomes Through Changes of Parental Input

In Table 2, descriptive statistics of measure of change over time in parental and child’s measures for the intervention and the control group are summarized. The results of Pearson’s correlation analyses testing the associations between measures of change of child’s and parental speech from pre- to post-intervention assessment are reported in the Supplementary Materials (see Supplementary Table 3). Almost every measure of change in parental speech—except for MLU—resulted positively and significantly associated to changes in child’s word types, tokens, and MLU.

Subsequent analyses focused on the indirect effects of parent-implemented language intervention on child’s speech changes in types and tokens production and MLU through changes in parental input, namely parents’ use of total responses, expansions, and amount of talking over reading during sessions (see Table 3 for the results of mediation analyses). Models, in which the indirect effect of the intervention via parental speech modification were significant, are also reported in the Supplementary Material (see Supplementary Figure 2).

**TABLE 3:** Estimated coefficients (unstandardized B and standardized coefficients a, b, c, c’) for mediation model of changes in parental use of total responses, expansions, and amount of talking over reading.

		**Total effect**				**Direct effect**				**Indirect effect**					
		**B**	**c**	**SE**	**95% CI**	**B**	**c’**	**SE**	**95% CI**	**B**	**a**	**b**	**ab**	**Boot SE**	**Boot 95% CI**
**Model**															
**Δ Total responses**															
	**Δ child’s speech**														
I	Types	7.61	0.27	4.06	[−0.58, 15.79]	3.64	0.53	3.62	[−3.65, 10.94]	3.96	0.55	0.53	0.29	2.56	[−0.68, 9.48]
II	Tokens	8.31	0.62	4.02	**[0.20, 16.41]**	4.24	0.32	3.53	[−2.88, 11.37]	4.06	0.55	0.55	0.30	2.48	[−0.61, 9.27]
III	MLU	8.87	0.66	3.90	**[0.99, 16.74]**	7.04	0.52	3.97	[−0.97, 15.04]	1.54	0.55	0.24	0.13	1.71	[−0.68, 5.82]
**Δ Expansions**															
	**Δ Child’s speech**														
IV	Types	7.61	0.57	4.06	[−0.58, 15.79]	2.21	0.16	3.65	[−5.17, 9.58]	5.40	0.70	0.58	0.41	2.91	**[0.84, 12.15]**
V	Tokens	8.30	0.62	4.02	**[0.20, 16.41]**	3.88	0.29	3.86	[−3.91, 11.66]	4.43	0.70	0.47	0.33	2.61	**[0.47, 10.46]**
VI	MLU	8.87	0.66	3.90	**[0.99, 16.74]**	8.30	0.62	4.20	[−0.18, 16.79]	0.57	0.70	0.06	0.04	1.73	[−2.51, 4.75]
**Δ Talking over reading**															
	**Δ Child’s speech**														
VII	Types	7.61	0.57	4.06	[−0,58, 15.79]	3.21	0.24	3.83	[−4.52, 10.93]	4.40	0.68	0.48	0.32	2.49	**[0.36, 10.12]**
VIII	Tokens	8.31	0.62	4.02	**[0.20, 16.41]**	3.42	0.25	3.65	[−3.94, 10.77]	4.89	0.68	0.53	0.36	2.62	**[0.54, 11.01]**
IX	MLU	8.87	0.66	3.90	**[0.99, 16.74]**	6.21	0.46	3.99	[−1.85, 14.27]	2.67	0.68	0.29	0.20	1.82	[−0.13, 6.83]

*Significant results are highlighted in bold.*

The first set of models (I, II, III) assessed indirect effects of the parent-implemented intervention on child’s changes in word types (I), tokens (II) and MLU (III) through parental changes in the use of total responses (see Table 3). All models yielded no significant direct and indirect effects, indicating the absence of significant mediation effects of changes in the parental use of total responses on the association between intervention and child’s progresses in word types, tokens and MLU.

The second set of models considered parental use of expansions as a mediator between intervention and child’s changes in word types (IV), tokens (V), and MLU (VI) (see Table 3 and Supplementary Figure 2). In model IV the indirect effect of the intervention on child’s changes in the production of word types resulted significant, whereas the direct effect was not. This implies that changes in the use of expansion by parents totally mediate the effect of the intervention on child’s gain in word types. Model V yielded similar results, with the intervention significantly influencing changes in the production of word tokens via changes in parental use of expansions. No indirect effects of the intervention *via* parental expansions were found in model VI including child’s MLU as the dependent variable.

The third set of analyses took in exam the indirect effect of intervention on child’s changes in word types (VII), tokens (VIII), and MLU (IX) through changes in the amount of parental talking over reading (see Table 3 and Supplementary Figure 2). In model VII and VIII the indirect effects of intervention on child’s changes in word types and tokens through changes in the amount of parental talking over reading were statistically significant. Again, no significant direct and indirect effects of the intervention were observed in the model predicting child’s MLU through amount of talking over reading. As a proof of concept, all models were further run using child’s measures as mediators and parental outcomes as independent measures. No significant indirect effects resulted from these analyses.

## Discussion

The present study aimed to investigate whether a parent-implemented language intervention targeted at parents of late-talkers might enhance the linguistic environment these children are exposed to through parental speech. We were particularly interested in evaluating which structural and functional features of parental speech could best benefit from an intervention based mainly on dialogic reading and focused stimulation techniques. This study further aimed to evaluate whether the intervention was effective in fostering children’s advances in language development and, more importantly, whether these effects were direct or mediated by modifications in parental speech features, consistently with the cascading effects of parent training model (Roberts and Kaiser, 2012; Heidlage et al., 2020).

### Intervention Effects Through Changes in Parental Speech

The study results contributed with novel findings to the literature, showing that the parent-implemented language intervention was effective in supporting children’s growth in expressive lexicon—with a significant increase in children’s lexical diversity (word types) and rate (word tokens)—indirectly, i.e., via the enhancement of functional features of parental speech. Children’s speech during parent-child interaction increased in terms of amount of words produced whereas parents demonstrated a growth in their responses to their child’s verbal attempts particularly by expanding them, adding extra verbal material able to provide the child with new attributes with respect to the expressed original meaning (e.g., the child says “hat” and the parent replies “that’s a very nice hat!”). Parents in the intervention group also showed better dialogic reading skills, spending less time reading to their children and more time conversating with them. The assessment of the direct and indirect effects of the intervention on children’s expressive skills indicated that the intervention positively affected parents’ use of expansions of children’s verbal initiatives and parents’ dialogic reading skills which, in turn, positively influenced children’s increase in the spontaneous use of word tokens and types. Although parent-implemented interventions assume that children’s difficulties in language development can be sustained by improving the quality of their linguistic environment, through changes in the parental input, studies documenting this cascading process are scant as most of the empirical work in this area only investigated a part of this process, namely the effects of intervention on children’s gains in language development. Studies addressing whether parent-implemented language interventions increased parents’ use of language and conversational strategies supporting language development are rare, as documented by the recent meta-analysis by Heidlage et al. (2020) that reported only five studies investigating this aspect. A recent work contributing to this debate (Kruythoff-Broekman et al., 2019) found that the parent-implemented Target Word program was effective in increasing parents’ communicative interaction with their children and in decreasing those behaviors aimed at putting pressure on their children and that this latter reduction, in turn, resulted associated to children’s progresses in expressive vocabulary and syntax. Unlike the present study, Kruythoff-Broekman et al.’s (2019) findings were not based on mediation analyses but on correlational models and no direction was tested, i.e., whether changes in parental intrusive behaviors might be a result of children’s gains in language development or vice versa.

In our study, parents participating to the “Oltre il libro” intervention exhibited relevant changes in the way they verbally interact with their children, compared to the parents in the control group. Parents receiving the intervention increased their total responses to their children’s verbal initiatives and, among total responses, used a greater amount of utterances intended at expanding their children’s utterances. A positive impact of parent-implemented interventions on parental responsiveness and use of expansions was suggested by Roberts and Kaiser’s (2011) meta-analysis and the more recent Heidlage et al. (2020), although both commented on the lack of strength of their findings due to the paucity of data supporting this conclusion. Our study contributed to reinforcing this finding, emphasizing the role of parent-implemented intervention in stimulating the parental use of total responses and expansions.

An increase of responses contiguous to children’s verbal attempts might be determinant for language learning as children—given the temporal connection between their initiatives and parental replies—can more easily make connections between labels and referents available in the context (Tamis-LeMonda et al., 2014). However, in our data, the increase in the use of total responses to children’s verbal attempts failed to mediate the effect of the intervention on children’s gains in their lexical and grammatical skills. This finding may be explained by considering that responses’ contiguity, if not accompanied by semantic contingency, can expose children to contents unrelated to their verbal initiatives, thus not immediately useful for their word learning (Tamis-LeMonda et al., 2014).

Different findings were observed regarding the increase of parental expansions which resulted as a significant mediator of the effects of the intervention on children’s advances in lexical diversity (word types) and rate (word tokens). Through expansions, children are provided not only with the repetition of their own verbal production—a feedback mechanism that confirms children their intended meaning and provides them with a phonologically correct version of the production—but they are further exposed to new data as syntactic information is added to children’s original verbal production. Moreover, this added material is likely to be semantically contingent to children’s verbal attempt, helping them to refine and expand their knowledge about the word and its meaning (Taumoepeau, 2016). Studies addressing both children with typical language development and late talkers showed that parental use of expansions contributed to children’s improvement in language development measures (Girolametto et al., 1999, 2002; Levickis et al., 2014, 2018; Taumoepeau, 2016). Positive associations between the use of expansions by parents and advances in language development of their late-talking children were observed when children’s language outcomes were assessed either with standardized tools, as in Levickis et al. (2014), or with direct observation of children’s spontaneous speech, as in the study of Girolametto et al. (2002).

Besides the significant improvements in the use of total responses and expansions, parents participating in the intervention also showed a significant increase in the talking over reading measure when compared to parents in the control condition. With this measure we intended to capture a parental dialogic reading style, as spending more time talking to the child—using prompts and connections to the child’s experiences and wh-questions to elicit a communicative exchange—rather than reading aloud without including the child, represented one of the aspects modeled by the intervention. In this sense, the intervention positively affected parents’ dialogic reading that used less verbatim reading of the text engaging their children in more verbal interaction over the shared books, and this change, in turn, favored children’s increased use of word types and tokens. Our conclusion is in line with literature findings assessing the effects of dialogic parent-child book reading interventions in promoting children’s language and literacy outcomes (Mol et al., 2008; Flack et al., 2018). A meta-analysis by Mol et al. (2008) highlighted that dialogic reading interventions are successful in fostering children’s expressive vocabulary with younger children—preschoolers vs. kindergarteners—gaining the best out of these programs. Book reading interventions also resulted beneficial for children with limited expressive vocabularies, as in Hargrave and Sénéchal (2000) study that compared children receiving dialogic reading vs. regular reading interventions, and as in Tsybina and Eriks-Brophy (2010) addressing bilingual preschoolers with slow expressive vocabulary development.

Concerning the structural features of parental speech input our study indicated the absence of significant changes due to the intervention, even if adjustments of the input to match children’s language abilities were modeled. Parents taking part in the intervention did not exhibit modifications in the lexical diversity (word types) and rate (word tokens) of the utterances directed to their children during the book sharing interaction nor in their speech grammatical complexity (MLU). Previous works evaluating modifications in parental speech due to parent-implemented interventions mostly took into exam changes in parental responsivity and use of conversational strategies. With regard to structural features of the input results are mixed. Girolametto et al. (1996a) found that the HPP was effective in decreasing maternal input complexity: mothers enrolled in the intervention used a slower rate of words per minute and shorter utterances when assessed at post-intervention. Differently, Roberts and Kaiser’s (2011) meta-analysis, reported a lack of significant effects of parent-implemented interventions on parents’ rate of speech. Overall, results regarding structural modifications of parental input are scant and inconsistent, and further studies are needed to shed light on this issue.

Summing up, our study brings new evidence of the effectiveness of parent-implemented interventions in affecting late talkers’ growth in expressive lexicon—diversity (word types) and rate (word tokens)—highlighting how these effects are mediated by significant modifications in parental use of expansions and dialogic reading skill. Some limits and strengths of the study are discussed below.

### Limitations and Strengths of the Study

Some limitations must be considered. The first regards the lack of randomization in the assignment of the participants to the study’s conditions. Participants’ inclusion in the intervention or control conditions was performed with a convenience method which might lead to a selection bias regarding differences in motivation and readiness to endorse the intervention and to baseline differences in the target measures. While this latter issue was resolved by controlling for pre-assessment differences, the former cannot be really ruled out. Furthermore, the study sample was limited and, for this reason, we should be cautious in generalizing our findings to the late-talkers’ population. Moreover, participants in the intervention and control group differed for their child-care center attendance, as more children in the intervention were enrolled in a child-care program. The opportunity of social engagement with peers and educators in such context could be beneficial for late-talkers’ language development, as suggested by Chen et al. (2020) with regard to a peer effect for language development in preschoolers, although no data are available on this topic concerning the role of child-care center attendance on younger children. A second limit concerns the lack of a long-term follow-up assessing the maintenance of the effects of the intervention. Studies addressing long-term effects of parent-implemented interventions are mixed in their findings documenting both long-lasting effects of interventions on children’s language scores and abilities and a lack of long-term effects in other cases (Buschmann et al., 2015; Kruythoff-Broekman et al., 2019). Ongoing monitoring of late talkers’ language development receiving parent-implemented interventions is relevant to determine the timing and dose of such programs. A final limitation- which was mainly due to our sample size—regards the lack of examination of the role of birth condition in moderating the effects of the intervention on parental and child’s speech outcomes. As preterm birth is considered a risk factor both for parent-child interaction (Bilgin and Wolke, 2015; Cambonie et al., 2017) and child’s language development (Sansavini et al., 2010), it would be interesting to examine in future studies whether or not it might play a moderator role in this intervention. In this study, this variable was included as a covariate so that our results can apply to both low-risk preterm and full-term child-parent dyads.

The study also presents some relevant strengths. The first regards the nature of speech data collected as both parental and children speech measures were computed through a fine-grained analysis of the sessions’ transcripts using a set of software specifically developed for such a complex material. Differently from other works in the field parents’ total responses, reformulations and reading style were computed directly from the transcript and not assessed by using rating scales or global evaluations (Kruythoff-Broekman et al., 2019), and the same occurred for the analysis of child’s spontaneous speech (Zuccarini et al., 2020). Another relevant strength regards the selected data analysis strategy and, in particular, the inclusion of models testing the direct and indirect effects of the parent-implemented intervention on children’s advances in lexical and grammatical skills. Although this kind of analysis is neither new nor scarcely used, this is the first time that such analysis is used to test parent-implemented intervention effectiveness.

### Implications for Practice

The main implication concerns the effectiveness of parent-implemented language intervention in supporting the language development of children showing a language delay. Although effects of such interventions are largely documented, our study shed light on the mechanisms of such effectiveness showing that modifications in parent use of expansions and dialogic reading abilities have cascading effects on children’s vocabulary growth and rate of speech. These results point to emphasize those features of parental training directed at enhancing functional features of parental speech input rather than at modifying structural features of such register. These findings can also inform other kinds of intervention, such as those delivered by child-care programs, and lead to the identification of language activities that can support language development of children attending child-care centers and preschool with poor language skills or at risk of delays.

## Conclusion

In this study, a parent-implemented intervention based on two main techniques, i.e., dialogic book reading and focused stimulation, was administered through six sessions during a two-month-period. This low-dosage intervention resulted in significant modifications of parental speech and children’s expressive outcomes at post-intervention assessment; our results showed that modifications in parents’ use of expansions and dialogic reading abilities mediated the effects of the intervention on children’s increase in lexical diversity and rate of words during a parent-child interaction. Our findings call for greater attention not only to children’s and parental outcomes but also to the intervention mechanisms promoting late-talkers’ linguistic advances and stress that the parental ability to engage in a conversation over a shared focus—the book’s storyline in our case—and respond contingently to child’s verbal attempts should be central in designing ecological and effective parent-implemented language interventions.

## Data Availability Statement

The dataset presented in this article is not readily available because it includes sensitive information about minors with developmental vulnerabilities. Requests to access the dataset should be directed to corresponding authors.

## Ethics Statement

The studies involving human participants were reviewed and approved by the Bologna Health Authority’s Independent Ethics Committee (numbers of formal approval documents: EM 194/2017/U_ and EM 193–2018_76/2013/U/Sper/AOUBo). Written informed consent to participate in this study was provided by the participants’ legal guardian/next of kin.

## Author Contributions

CS, AS, AG, and MZ: conceptualization and methodology. MZ: data collection. AA and LC: medical aspects of methodology and medical data collection and supervision. CS: data transcription, data analysis. CS and MZ: data coding and data curation. CS, AS, and AG: writing—original draft and review and editing. AS and AG: funding acquisition. AS: project administration and supervision. All authors have read and approved the manuscript.

## Conflict of Interest

The authors declare that the research was conducted in the absence of any commercial or financial relationships that could be construed as a potential conflict of interest.

## Publisher’s Note

All claims expressed in this article are solely those of the authors and do not necessarily represent those of their affiliated organizations, or those of the publisher, the editors and the reviewers. Any product that may be evaluated in this article, or claim that may be made by its manufacturer, is not guaranteed or endorsed by the publisher.

## Supplementary Material

Supplementary Figure 1 Flow diagram of late talkers whose parents participated in the study.

Supplementary Figure 2 Parental changes in expansions (models IV and V) and talking over reading (models VII and VIII) as mediators of the parent-implemented intervention effect on child’s speech types and tokens.

Supplementary Table 1 Clinical and Perinatal Characteristics of the Low-Risk Preterm Children.

Supplementary Table 2 Descriptive statistics of parental and child’s speech measure at the pre-intervention assessment for the intervention and the control group. The table also summarizes the results of multivariate MANCOVAs addressing groups differences at pre-intervention.

Supplementary Table 3 Pearson’s correlation coefficients among study’s variables of change in parental and child’s speech.
