# Supplementary material for: The Effects of a Parent-Implemented Language Intervention on Late-Talkers’ Expressive Skills: The Mediational Role of Parental Speech Contingency and Dialogic Reading Abilities
Source: Front Psychol. 2021 Sep 9;12:723366. doi: 10.3389/fpsyg.2021.723366 (PMC8459088; doi:10.3389/fpsyg.2021.723366)
Supplement: Supplementary Figure 1 — Flow diagram of late talkers whose parents participated in the study. [file Data_Sheet_1.pdf]

Supplementary Materials

Table S1: Clinical and Perinatal Characteristics of the Low-Risk Preterm Children

	Low-Risk Preterm Children (n = 17)
Clinical and Perinatal Characteristics	
Gestational Age (weeks), Mean (SD)	34.17 (2.30)
Birthweight (grams), Mean (SD)	1996 (525.30)
Length of Stay in Hospital (days), Mean (SD)	20.12 (41.47)
Gender (Female), *n* (%)	6 (35.3)
Firstborn, *n* (%)	10 (58.8)
Twins, *n* (%)	8 (47.0)
Otitis Media, *n* (%)	1 (5.8)
Family History of Language and/or Learning Disorders (LLD), *n* (%)	5 (29.4)
Caesarean Section, *n* (%)	15 (88.2)
SGA, *n* (%)	5 (29.0)
IVH I/II, *n* (%)	0 (0.0)
MV, *n* (%)	1 (5.9)
RDS, *n* (%)	7 (41.2)
Apnea, *n* (%)	1 (5.9)
BDP, *n* (%)	0 (0.0)
Sepsis, *n* (%)	1 (5.9)
ROP I/II, *n* (%)	0 (0.0)
Hyperbilirubinemia with Phototherapy, *n* (%)	9 (52.9)

SGA: Small for gestational age, infants with a birthweight < 10th percentile for gestational age; IVH I/II: intra-ventricular hemorrhage originating within the subependymal germinal matrix filling less than 10% (I grade) and 50% (II grade), respectively, of the ventricular area on parasagittal view; MV: mechanical ventilation; RDS: respiratory distress syndrome, acute illness coming on within 4–6 h of delivery, characterized clinically by respiratory rate ≥ 60/min, dyspnea and respiratory distress; Apnea: more than four episodes of apnea/hour or more than two episodes of apnea/hour if ventilation with a bag and mask was required; BDP: bronchopulmonary dysplasia, needing both supplemental oxygen for ≥ 28 days and at 36 weeks of post-conception age; Sepsis: presence of a positive blood culture and/or clinical and laboratory signs; ROP I/II: retinopathy of prematurity, vasoproliferative retinopathy resolved without a specific therapy before the presumed date of birth; Hyperbilirubinemia with phototherapy: hyperbilirubinemia needing phototherapy according to the criteria proposed by Gomella et al. (2004).

Table S2: Descriptive statistics of parental and child's speech measure at the pre-intervention assessment for the intervention and the control group. The table also summarizes the results of multivariate MANCOVAs addressing groups differences at pre-intervention.

	Pre-intervention assessment		Multivariate Tests			Univariate Tests		
	Intervention (*n* =24)	Control (*n* = 22)	*F*(1,43)	*p*	partial η2	*F*(1,43)	*p*	partial η2
Parental Speech - Structural Features			0.71	0.554	0.049			
Types	202.92 (38.44)	182.32 (47.91)				0.80	0.377	0.018
Tokens	603.13 (151.59)	563.55 (201.46)				0.03	0.868	0.001
MLU	3.25 (0.42)	3.14 (0.51)				0.64	0.428	0.015
Parental Speech - Functional Features			0.99	0.435	0.113			
Total Responses	96.92 (55.57)	108.05 (57.69)				1.63	0.209	0.036
Exacts	3.63 (3.69)	2.95 (3.40)				0.01	0.913	0.000
Reductions	0.29 (0.69)	0.60 (1.26)				0.01	0.983	0.001
Expansions	4.29 (4.92)	2.64 (3.26)				0.11	0.744	0.003
Talking over reading (%)	0.69 (0.14)	0.71 (0.17)				1.05	0.311	0.024
Child's speech			1.48	0.223	0.098			
Types	9.28 (9.09)	6.04 (4.95)				0.10	0.750	0.002
Tokens	6.11 (8.22)	10.17 (10.84)				2.06	0.158	0.046
MLU	1.05 (0.26)	0.98 (0.33)				0.01	0.932	0.000

Table S3: Pearson's correlation coefficients among study's variables of change in parental and child's speech

	Types	Tokens	MLU	Total Responses	Exacts	Reductions	Expansions	Talking over Reading	Child's Types	Child's Tokens	Child's MLU
Δ Parental Speech											
Types	1										
Tokens	.756**	1									
MLU	.416**	.231	1								
Total Responses	.112	.509**	-.269	1							
Exacts	.173	.340*	-.216	.572**	1						
Reductions	.158	.441**	-.053	.537**	.283	1					
Expansions	.269	.480**	-.09	.598**	.666**	.277	1				
Talking over reading	.631**	.829**	.141	.533**	.275	.509**	.377**	1			
Δ Child's Speech											
Types	.300*	.417**	-.07	.557**	.479**	.456**	.585**	.521**	1		
Tokens	.336*	.456**	-.067	.588**	.342*	.499**	.482**	.576**	.885**	1	
MLU	.148	.230	-.117	.419**	.077	0.489**	.187	.344*	.567**	.618**	1

** *p* <.001; * *p*<.01

Figure S1

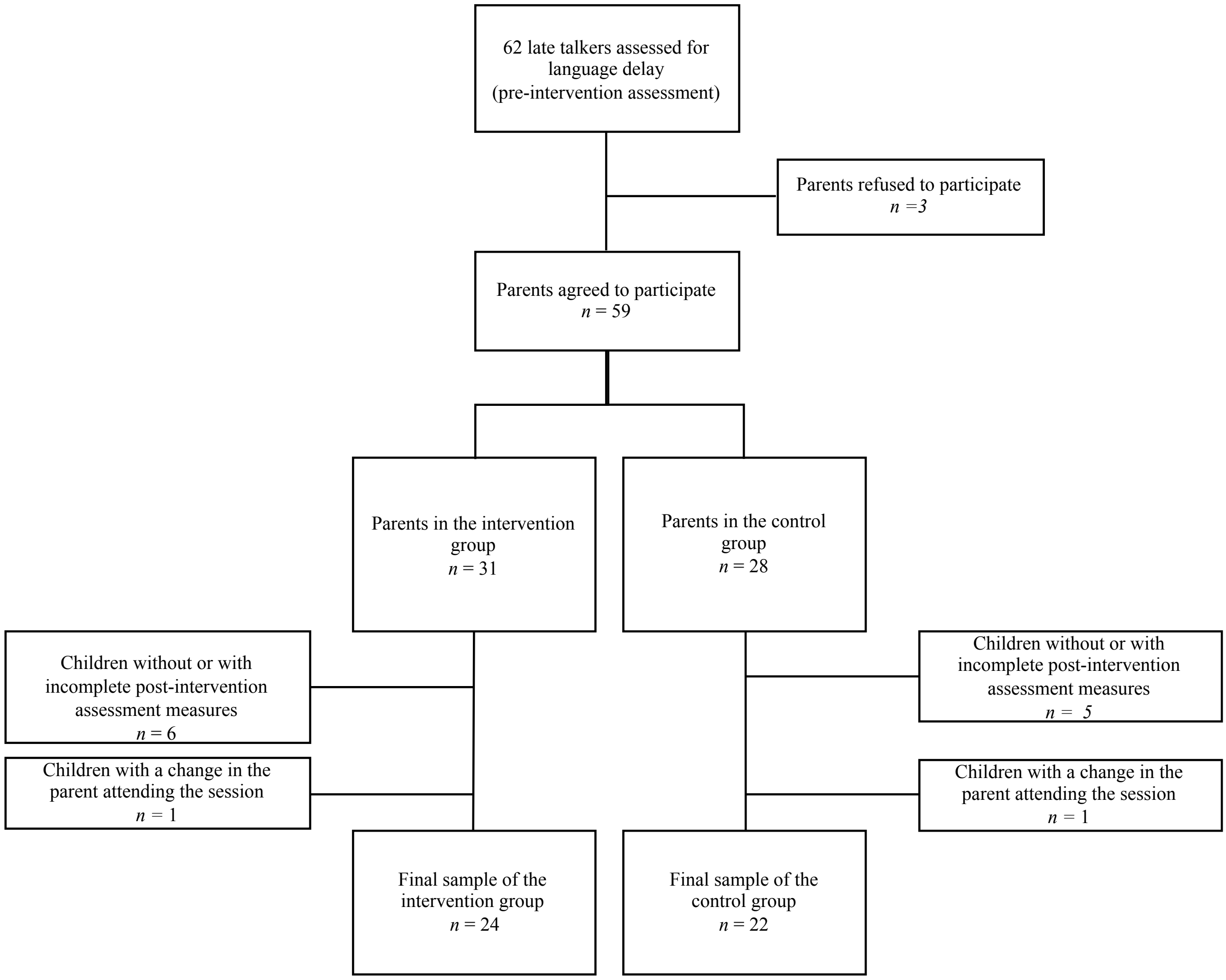
62 late talkers assessed for language delay (pre-intervention assessment)
Parents refused to participate n =3
Parents agreed to participate n = 59
Parents in the intervention group n = 31
Parents in the control group n = 28
Children without or with incomplete post-intervention assessment measures n = 6
Children without or with incomplete post-intervention assessment measures n = 5
Children with a change in the parent attending the session n = 1
Children with a change in the parent attending the session n = 1
Final sample of the intervention group n = 24
Final sample of the control group n = 22

Figure S2

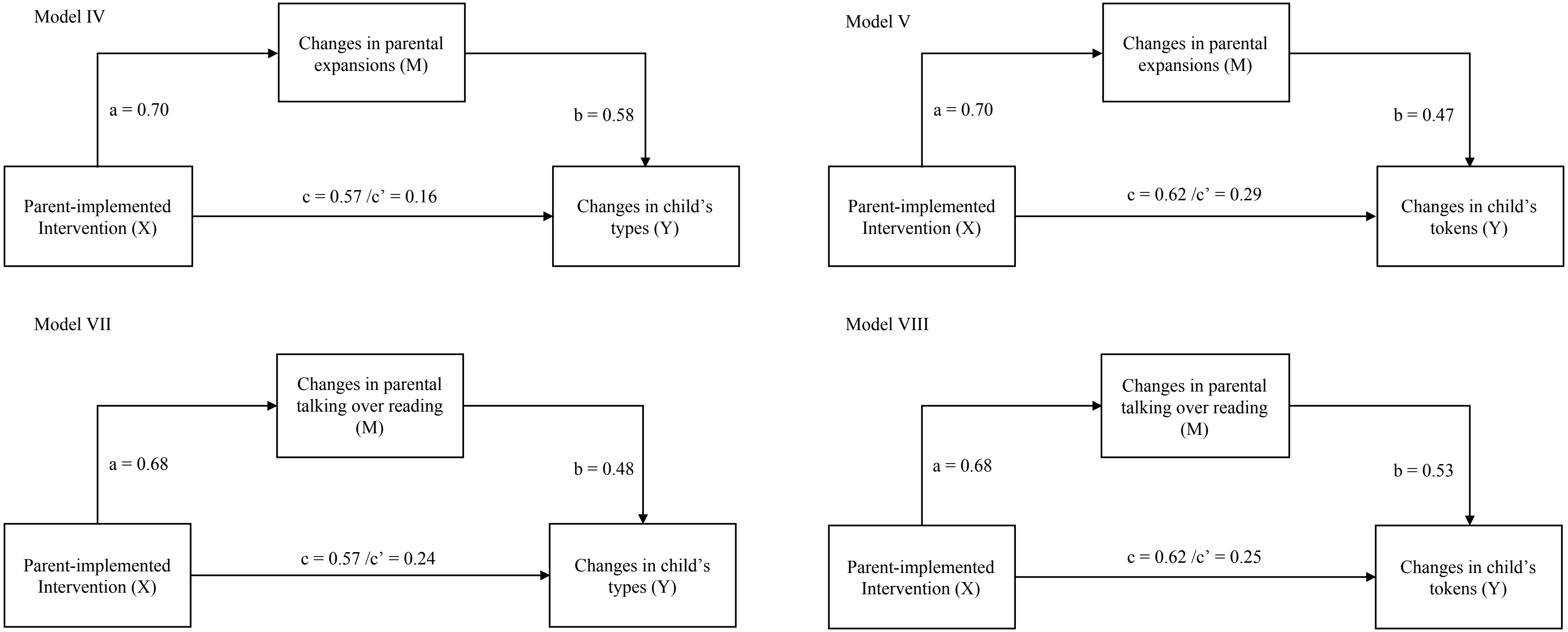
Model IV
Changes in parental expansions (M)
a = 0.70
b = 0.58
Parent-implemented Intervention (X)
c = 0.57 /c' = 0.16
Changes in child's types (Y)
Model V
Changes in parental expansions (M)
a = 0.70
b = 0.47
Parent-implemented Intervention (X)
c = 0.62 /c' = 0.29
Changes in child's tokens (Y)
Model VII
Changes in parental talking over reading (M)
a = 0.68
b = 0.48
Parent-implemented Intervention (X)
c = 0.57 /c' = 0.24
Changes in child's types (Y)
Model VIII
Changes in parental talking over reading (M)
a = 0.68
b = 0.53
Parent-implemented Intervention (X)
c = 0.62 /c' = 0.25
Changes in child's tokens (Y)
